# Preferences, Partners, and Parenthood: Linking Early Fertility Desires, Marriage Timing, and Achieved Fertility

**DOI:** 10.1007/s13524-020-00927-y

**Published:** 2020-11-12

**Authors:** Natalie Nitsche, Sarah R. Hayford

**Affiliations:** 1 Max Planck Institute for Demographic Research, Rostock, Germany; 2 Department of Sociology and Institute for Population Research, The Ohio State University, Columbus, OH USA

**Keywords:** Fertility, Fertility desires, Marriage, Education

## Abstract

In the United States, underachieving fertility desires is more common among women with higher levels of education and those who delay first marriage beyond their mid-20s. However, the relationship between these patterns, and particularly the degree to which marriage postponement *explains* lower fertility among the highly educated, is not well understood. We use data from the National Longitudinal Survey of Youth 1979 cohort to analyze differences in parenthood and achieved parity for men and women, focusing on the role of marriage timing in achieving fertility goals over the life course. We expand on previous research by distinguishing between entry into parenthood and average parity among parents as pathways to underachieving, by considering variation in the impact of marriage timing by education and by stage of the life course, and by comparing results for men and women. We find that women with a bachelor’s degree who desired three or more children are less likely to become mothers relative to women with the same desired family size who did not attend college. Conditional on becoming mothers, however, women with at least a bachelor’s degree do not have lower completed family size. No comparable fatherhood difference by desired family size is present. Postponing marriage beyond age 30 is associated with lower proportions of parenthood but not with lower parity among parents. Age patterns are similar for women and men, pointing at social rather than biological factors driving the underachievement of fertility goals.

**Electronic supplementary material:**

The online version of this article (10.1007/s13524-020-00927-y) contains supplementary material, which is available to authorized users.

## Introduction

Aggregate fertility rates have settled at well below replacement in many advanced industrialized countries. At the same time, the two-child family ideal has remained strong (Sobotka and Beaujouan 2014). *Unrealized fertility*, or the underachievement of fertility aspirations, has thus become a common occurrence (Bongaarts 2001; Casterline 2019) and can have meaningful implications for individuals’ well-being as well as for population growth rates (Casterline and Han 2017; McQuillan et al. 2003, 2012). Even in contexts where desired and achieved fertility match at the aggregate level, such as the United States, there is substantial variation in who meets fertility goals on the individual level (Hagewen and Morgan 2005; Harknett and Hartnett 2014). In particular, underachievement of fertility intentions occurs most often among college-educated women, at least in the United States (Morgan and Rackin 2010; Quesnel-Vallée and Morgan 2003).

Here, we seek to understand the phenomenon of underachieving fertility desires in the United States by breaking down this process along multiple axes and by analyzing marital timing as one key mechanism that may produce underachieving. Using data from the National Longitudinal Survey of Youth 1979 (NLSY79), we offer a two-step analytical approach. First, we examine who underachieves and to what extent. We distinguish between unachieved parenthood and underachieved parity among parents and describe these processes separately by desired parity and completed education. Second, we investigate the impact of marital timing for subsequent parenthood status and number of children among parents at age 43, using stepwise modeling and mediation analysis. We furthermore describe how these processes differ for men and women.

## “Underachieving”

In advanced industrialized societies, many men and women reach the end of their childbearing years with fewer children than they desired earlier in life. This phenomenon, measured at the aggregate or the individual level, is referred to as the *underachievement* of fertility goals. People change their fertility goals over time, often in response to life events (e.g., Gemmill 2019; Gray et al. 2013; Hayford 2009; Heiland et al. 2008; Iacovou and Tavares 2011; Rybińska and Morgan 2018), so inconsistencies between early desires, expectations, or intentions and later outcomes may represent evolving goals rather than failures in achieving these goals. Still, considering the difference between desired and achieved trajectories can shed light on possible sources of constraint and recalibration, and measuring underachievement provides a big-picture summary of the underlying life course processes.

Previous studies have drawn on data from cohort panel surveys in the United States (NLSY79) and the United Kingdom (National Child Development Study (NCDS)) to compare early fertility desires and intentions with achieved fertility (Berrington and Pattaro 2014; Morgan and Rackin 2010; Quesnel-Vallée and Morgan 2003). Roughly 43% of women and 34% to 36% of men achieve their exact intended parity in the NLSY79 (Morgan and Rackin 2010) and in the NCDS (Berrington and Pattaro 2014), respectively. Underachieving is much more common than overachieving, particularly among men, the more highly educated, and those intending larger families (Berrington and Pattaro 2014; Morgan and Rackin 2010; Quesnel-Vallée and Morgan 2003).

These studies have defined underachieving as the net or gross error: in other words, the discrepancy between desired or intended lifetime parity at ages 16–24 and achieved parity after age 40. This definition has several limitations. First, it conflates desires and outcomes. Individuals with higher desired parity need to have many children to meet their fertility desires or to overachieve, but a single birth means overachieving for those desiring no children. Numerically speaking, underachieving is thus more likely to occur among those desiring larger families even though, on average, they will have more children. Second, relying on the discrepancy measure means that we know little about whether underachieving comes about via the lack of parenthood altogether, lower parity among parents, or both and to what extent. The same is true for the factors mediating fertility desires and childbearing behavior, such as education and union histories. Third, as a result of these first two limitations, it remains unclear whether a greater likelihood of underachieving—for example, among those who never marry or the highly educated—may perhaps simply hinge on systematically larger desired family sizes among these individuals early in life.

Examining parity-specific patterns of underachievement can help address these limitations. For the United Kingdom, Berrington and Pattaro (2014) differentiated underachieving separately by intended parity. They showed that highly educated women intending larger families are more likely both to underachieve and to overachieve than their less-educated counterparts, while highly educated women intending to bear two children are not more likely to overachieve. In the United States, parity-specific underachievement has not been studied. However, Musick et al. (2009) pointed to the importance of differential levels of overachievement of women who intended no children for explaining education differences in fertility. Less-educated women in the United States are more likely to overachieve early fertility intentions, largely via mistimed or unwanted births (Musick et al. 2009). If those who did not want or intend children are more likely to become mothers among the lower- than the higher-educated, this pattern will affect education-specific summary measures of underachieving.

## Partners, Marriage, and Underachieving

The literature suggests three primary pathways into underachieving fertility desires. First, involuntary infertility may make it impossible to become a parent or to have the desired number of children. Because of data limitations, the impact of medical infertility is rarely studied in population data, although it is often discussed as an unmeasured residual mechanism. The second is the development of competing preferences—such as aspirations for education, employment, or leisure—that lead people to change their desired family size (Ajzen 2005; Berrington and Pattaro 2014; Blair-Loy 2009). The third is the repeated postponement of parenthood that eventually leads to conflict with biological and social age limits for childbearing (Billari et al. 2007; Gemmill 2019; Rybińska and Morgan 2018). In practice, these pathways may intersect—for example, if postponed parenthood due to education and labor market activities leads to changed preferences or age-related infertility. Parenthood postponement due to work-family incompatibilities may operate more prominently among women than among men because women need to interrupt or rearrange employment before and after giving birth and often will be primary caregivers and take on more housework after children are born (Bianchi et al. 2012; Nitsche and Grunow 2016).

The postponement of coresidential or marital unions can be another aspect leading to running out of time (Casterline and Han 2017), but it has received less attention than education or labor market conflicts as a unique driver of underachievement. Empirically, however, union formation status and trajectories have been shown to be a central predictor of underachieving, and particularly of education gradients in underachieving (e.g., Berrington and Pattaro 2014; Morgan and Rackin 2010; Mynarska et al. 2015). In this study, we consider the implications of marriage delay and nonmarriage for underachieving early fertility desires, through both achieved parenthood and achieved parity, and for both men and women. Because of the substantial variation in nonmarital fertility rates across education levels in the United States, we consider variation by education in the role of marital timing in achieved fertility.

We focus on marriage because it is a central component in U.S. family systems. (Supplementary analyses, described in the upcoming Methods section, analyzed the impact of any coresidential union on fertility and found similar associations.) Marriage as an institutionalized form of partnership has long been a normative expectation for parenthood in the United States, and especially so among the highly educated and the cohorts represented by the NLSY79 (Cherlin 2009; Thornton et al. 2007). Thus, people may prefer to limit childbearing outside of marriage. If marriage is delayed until an age range where biological or social factors limit fertility, delayed marriage may result in forgone childbearing. Social norms around appropriate ages for childbearing, or worries about implications for the health of the offspring when pregnancy is delayed into a woman’s late 30s or 40s, may also lead to declines in birth rates among these age groups (Mynarska 2010; Van Bavel and Nitsche 2013).

## Hypotheses

We have two primary goals in this article, both largely descriptive. First, we seek to understand how underachieving early fertility goals varies by completed education and desired parity and to disentangle the roles of entry into parenthood and parity among parents in this variation. We do not have *a priori* hypotheses for this component of the analysis. Second, we analyze the role of delayed or foregone marriage in underachieving. This analysis does not speak to the sources of delayed marriage or nonmarriage, and we cannot identify whether behaviors are responding to marriage market conditions or are the result of deliberate choices. Rather, we seek to determine the role of marriage timing as a distinct driver of fertility behavior separate from fertility timing within marriage. Here, we propose several basic hypotheses in order to facilitate interpretation of results.

Overall, we expect later marriage to be associated with lower rates of entry into parenthood (Hypothesis 1 (H1)). Age limits for fertility have been perceived more strongly for women than men. As a result, marriage delay may impact men’s fertility less strongly than women’s fertility, and impacts may come at later ages. These differences may be exacerbated by gender differences in age at marriage (i.e., by the fact that husbands are generally older than their wives). We therefore expect the association between marriage timing and parenthood to be weaker and to take effect at older ages for men than for women (Hypothesis 1a (H1a)). We expect a delay of marriage to limit entry into parenthood more strongly among the college-educated than among the lower-educated, given that the most-educated tend to limit nonmarital fertility (Hypothesis 1b (H1b)).

Similarly, we hypothesize that later marriage will be linked to lower completed parity among those who become parents (Hypothesis 2 (H2)) and that declines will affect women more than men (Hypothesis 2a (H2a)). To the extent that differences in higher-order fertility are driven by the same social and biological age constraints as differences in first births, we may see impacts of marriage timing on parity at lower ages than impacts on parenthood. However, we expect to see relatively few education differences in the impact of marriage timing on parity (Hypothesis 2b (H2b)). By conditioning on first birth, we effectively limit our sample to those individuals who either have a marital first birth or who do not see marriage as a prerequisite to childbearing. Thus, we do not expect the stronger impact of marriage timing on parity for college-educated men and women that we hypothesize for entry into parenthood.

Finally, selection into earlier ages at marriage may take place based on fertility desires. We adjust for such selection by controlling for the desired number of children.

## Data, Sample, and Measurements

### Data and Sample

The data for our analyses come from the NLSY79, a U.S. panel study of individuals born between 1957 and 1964. A nationally representative sample was first interviewed in 1979, resurveyed annually until 1994, and thereafter surveyed biennially. The NLSY79 includes rich information on fertility, marriage, education, and labor market experiences as well as information on family background.

For our analyses, we select only those individuals aged 18 and younger at the first interview, yielding a sample size of 7,217 individuals: 3,673 men and 3,544 women. We impose this restriction to measure the desired number of children roughly at the same point in the life course for all respondents and to measure desired fertility before family formation has begun. In this cohort, about one-third of women have had their first birth by the age of 22 (Aughinbaugh and Sun 2016); hence, we exclude the sample of those aged 19–22 at first interview. We also limit the sample to individuals observed at or beyond age 43 at least once to obtain information on their achieved fertility. This leaves us with a sample size of 2,661 men and 2,642 women, reflecting a retention of 73% of the original sample of the specified age group. (About one-half of the sample attrition is from the military subsample or the economically disadvantaged non-Black/non-Hispanic subsample, both of which were dropped after 1984 and 1990, respectively.)

### Measurements

Our dependent variables are *parenthood status* and *number of children* ever born, measured at age 43. We choose age 43 as cutoff because fecundity appears to taper off relatively quickly after that age, but births around age 40 still can contribute substantively to fertility histories of women with higher educational attainment (Beaujouan and Sobotka 2017).

Our key predictors are completed education, the desired number of children at ages 14–18, and the age at first marriage. *Completed educational attainment* is measured as the highest reported level of schooling completed: did not graduate from high school, high school diploma, some college or associate’s degree, and bachelor’s degree or more education.

We measure the *desired number of children* in 1979 (“How many children do you want to have?”), collapsed into three categories: (1) zero children or one child (14.8%), (2) two children (44.4%), and (3) three or more children (40%). We choose this categorization because exploratory analyses indicate that variation from the strong norm of a two-child family is the strongest predictor of eventual fertility. Unfortunately, cell sizes are too small to examine those desiring no children and those desiring one child separately. To the extent that variation within these three categories is different at different education levels, this categorization may under- or overestimate true educational variation in achieving desired fertility. However, exploratory analysis (not shown) indicates that average desired parity *within* the categories 0/1 and 3 children or more is similar across education groups. Desired fertility measured early in the life course is a limited measure of a complex construct: goals for childbearing. For most adolescents, desired fertility does not represent a well-developed plan or a specific intention for behavior. Rather, desired family size may reflect an adolescent’s assessment of what constitutes a so-called good or normal family based on exposure to families they see around them, media representations, or cultural schemas (e.g., drawn from religious teachings; Bachrach and Morgan 2013). Still, this measure is predictive of future fertility, and comparing early desired fertility with later outcomes can shed light on how individuals think about childbearing and how life course experiences interact with desires to produce behavior.

*Marriage timing* is a categorical variable with six categories, indicating the age by which the first marriage occurred, measured in (approximately) five-year steps: by age 20, between 21 and 25, between 26 and 30, between 31 and 35, between 36 and 43, and later or never. We also conducted sensitivity tests using age at first coresidential union (cohabitation or marriage) instead of age at first marriage. Results from these supplemental analyses (available on request) were largely similar to the main results shown here, perhaps in part because cohabitation was relatively rare for these cohorts. Only one-fifth (19.5%) of respondents had an age at first coresidential union younger than the age at first marriage. For unions formed at older ages (after age 30), the magnitude of association is somewhat larger for coresidential unions than for marriage.

Furthermore, all models *control* for race (Black or White),^1^ Hispanic origin (yes or no), family structure at age 14 (living with both biological parents, yes or no), number of siblings, mother’s education (measured in four categories as for respondent’s education), and frequency of attending religious services in 1979 (measured on a scale from 1 to 6, representing six categories ranging from “never” to “more than once a week”). Models estimating parity also control for respondent’s age at first birth.

### Analytic Strategy

We estimate logistic regression models to predict parenthood status and generalized linear models (GLM) based on a Poisson distribution to predict achieved parity among parents. All models account for the aforementioned control variables. We present results from four model specifications. The first specification estimates parenthood and parity at age 43 by desired family size and education (interactive) without incorporating marital timing. These models provide a baseline assessment of education differences in achieved fertility by desired family size. The second set of models predicts parenthood and parity by marital timing, controlling for completed education and desired family size. The third set estimates parenthood/parity by marital timing allowing for an interaction between marital timing and completed education. The results of the second and third sets of models provide an overall estimate of how fine-grained marital timing is related to fertility at age 43, testing H1 and H2 as well as H1b and H2b. We compare results for men and women to test H1a and H2a.

Finally, the fourth set of models tests for the mediating effect of marital timing on the relationship between education-specific fertility desires and achieved parenthood and parity in two ways, further testing H1b and H2b. First, we repeat the first specification (parenthood and parity at age 43 by desired family size and education) and compare predicted values before and after adding the marital timing measurement to assess whether education differences in underachieving shrink or disappear when marital timing is controlled for. Because marital timing appears to mediate education and desired parity differences with regards to motherhood (not fatherhood or parity), we then use a more formal method to test for its mediating effect. We decompose the total effect of education-specific fertility desires into a direct effect and an indirect effect. The direct effect represents differences in achieved motherhood by (education-specific) fertility desires holding marital timing constant. That is, the direct effect is a counterfactual estimate of how much achieved motherhood would vary if marital timing did not differ across levels of education-specific fertility desires. The indirect effect describes the difference in achieved motherhood attributable to differences in marital timing across education-specific fertility desires. We estimate these models using the *ldecomp* command in Stata 15 (Buis 2010). Results of the mediation models are not formally shown but are mentioned in the Results section when relevant.

We present our findings using predicted values generated using the *margins* command in Stata 15. Predicted probabilities are shown in tables and figures. Regression coefficients for the first specification (baseline models) and the fourth specification (baseline models adding marital timing) are shown in the online appendix (Tables A–D). Regression coefficients for the other specifications are available upon request.

## Results

### Sample Description

Basic information on the distribution of men and women in our analytic sample across desired fertility and completed education categories, as well as the distribution of age at marriage, is presented in Table 1. In all completed education groups, for both men and women, desiring no children or one child is the least-frequent response. Desiring fewer than two children is relatively more common among lower education levels; for example, 17.5% of men and 21.3% of women without a high school education want fewer than two children, compared with 5.1% and 13.1%, respectively, of men and women with a four-year college degree. Desiring two children is the modal category for all education groups except for those with a bachelor’s degree or higher. Among both women and men in this group, desiring three or more children is most common (46.2% of women and 48.6% of men).

**Table 1:** Sample description, men and women aged 14–18 at first interview in the NLSY79

			By Educational Attainment							
	Analytic Sample		Men				Women			
	Men	Women	No High School Diploma	High School Diploma	Some College	Bachelor’s Degree or Higher	No High School Diploma	High School Diploma	Some College	Bachelor’s Degree or Higher
*N*	2,589	2,566	268	1,273	519	529	203	1,064	702	597
Educational Attainment (%)										
No high school diploma	10.4	7.9								
High school diploma	49.2	41.5								
Some college	20.1	27.4								
Bachelor’s degree or higher	20.4	23.3								
Desired Number of Children (%)										
0 or 1	11.8	17.3	17.5	14.4	9.6	5.2	21.3	18.9	17.3	13.1
2	44.8	44.2	39.2	45.1	45.6	46.2	43.6	46.7	43.6	40.7
3+	43.4	38.5	43.4	40.6	44.8	48.7	35.2	34.5	39.1	46.2
Age at Marriage (%)										
By 20	14.5	31.2	21.6	17.9	12.7	4.4	47.8	38.7	31.6	11.6
By 25	32.8	30.1	26.5	32.3	37.2	32.7	15.8	26.8	32.5	38.2
By 30	17.4	13.2	11.2	14.1	16.4	29.3	7.9	9.0	11.0	25.1
By 35	9.2	6.6	6.7	7.8	8.7	14.4	4.9	5.7	5.8	9.7
By 43	5.0	4.1	6.3	4.2	5.8	5.7	4.9	3.4	4.6	4.7
Later or never	21.2	14.7	27.6	23.7	19.3	13.6	18.7	16.4	14.5	10.7

*Notes:* The table presents data on men and women aged 18 or older at the first interview, observed at least once at ages 43 or older, with nonmissing data on key dependent and independent variables.

*Source:* National Longitudinal Survey of Youth, 1979 cohort.

### Achieving and Underachieving of Early Fertility Desires

#### Entry Into Parenthood

**Fig. 1:**
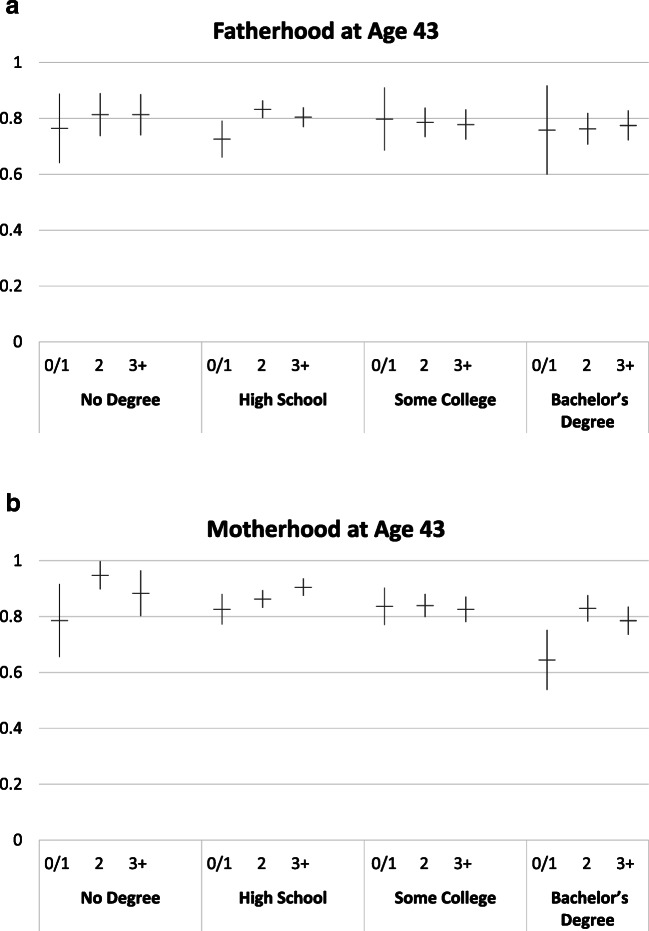
Predicted probabilities of fatherhood and motherhood at age 43 by desired number of children in adolescence and highest degree completed. Models control for race, Hispanic ethnicity, religiosity in 1979, family structure at age 14, number of siblings, and mother’s education.

Figure 1 shows predicted probabilities of parenthood based on the multivariate model described earlier, holding covariates at their mean value. Significant differences in the incidence of parenthood by the desired number of children emerge within and across education groups, and these differences are larger for women than for men. Among men, desiring no or one child is associated with a lower incidence of fatherhood only among those with a high school education; 73% of them will become fathers, significantly less than those desiring two children or three or more children (83% and 80%, respectively; see Table 2). Differences in parenthood between those desiring two children and those desiring three or more children are negligible among men. Comparing across education groups, differences in achieved parenthood by desired parity are generally small and not statistically significant, with one exception: desiring two children is associated with lower parenthood chances among men with a bachelor’s degree or more (76%) than those with a high school education (83%).

**Table 2:** Fatherhood at age 43, before and after controlling for first-marriage timing

		Baseline Model			Controlling for Marital Timing		
Education	Desired Number of Children	Estimate	CI Lower Bound	CI Upper Bound	Estimate	CI Lower Bound	CI Upper Bound
A. Predicted Probability of Fatherhood by Education and Desired Number of Children							
No high school diploma	0 or 1	.76	.64	.89	.83	.72	.94
	2	.81	.74	.89	.86	.78	.93
	3+	.81	.74	.89	.91	.86	.96
High school diploma	0 or 1	.73	.66	.79	.82	.76	.88
	2	.83	.80	.86	.88	.85	.91
	3+	.80	.77	.84	.87	.84	.90
Some college/associate’s degree	0 or 1	.80	.68	.91	.84	.73	.95
	2	.79	.73	.84	.83	.78	.88
	3+	.78	.72	.83	.84	.78	.89
Bachelor’s degree or higher	0 or 1	.76	.60	.92	.86	.74	.98
	2	.76	.71	.82	.83	.78	.88
	3+	.77	.72	.83	.83	.78	.88
	Contrast Tested	Baseline Model *p* Value			Controlling for Marital Timing *p* Value		
B. Tests of Significance for Selected Contrasts (*p* values based on chi-square tests)							
Within education, across desired family size							
No high school diploma	0 or 1 child vs. 2 children	.49			.67		
	3+ vs. 2 children	1.00			.18		
High school diploma	0 or 1 child vs. 2 children	.00 ***			.05 ^†^		
	3+ vs. 2 children	.23			.72		
Some college	0 or 1 child vs. 2 children	.85			.80		
	3+ vs. 2 children	.84			.84		
Bachelor’s degree or higher	0 or 1 child vs. 2 children	.96			.67		
	3+ vs. 2 children	.74			.90		
Within desired family size, across education							
No high school diploma vs. bachelor’s degree	0 or 1 child	.95			.71		
High school diploma vs. bachelor’s degree		.72			.58		
Some college vs. bachelor’s degree		.69			.85		
No high school diploma vs. bachelor’s degree	2 children	.32			.58		
High school diploma vs. bachelor’s degree		.03 *			.08 ^†^		
Some college vs. bachelor’s degree		.55			.97		
No high school diploma vs. bachelor’s degree	3+ children	.43			.04 *		
High school diploma vs. bachelor’s degree		.37			.18		
Some college vs. bachelor’s degree		.93			.96		

*Note: CI* = confidence interval.

^†^*p* < .10; *p < .05; ***p < .001

Desired parity is more strongly associated with achieved parenthood among women than among men. The prevalence of parenthood is lower for women desiring no or one child than for higher desired parities across all education groups except among women with some college education. However, these differences are statistically significant only for the highest and lowest education groups (Table 3).

**Table 3:** Motherhood at age 43, before and after controlling for first-marriage timing

		Baseline Model			Controlling for Marital Timing		
Education	Desired Number of Children	Estimate	CI Lower Bound	CI Upper Bound	Estimate	CI Lower Bound	CI Upper Bound
A. Predicted Probability of Motherhood by Education and Desired Number of Children							
No high school diploma	0 or 1	.79	.66	.92	.88	.79	.97
	2	.95	.90	1.00	.96	.92	1.00
	3+	.88	.80	.96	.90	.83	.98
High school diploma	0 or 1	.83	.77	.88	.86	.81	.91
	2	.86	.83	.89	.89	.86	.92
	3+	.91	.87	.94	.93	.90	.95
Some college	0 or 1	.84	.77	.90	.87	.81	.93
	2	.84	.80	.88	.86	.82	.90
	3+	.83	.78	.87	.84	.79	.89
Bachelor’s degree or more	0 or 1	.64	.54	.75	.66	.55	.78
	2	.83	.78	.88	.86	.81	.90
	3+	.79	.74	.84	.83	.79	.88
	Contrast Tested	Baseline Model *p* Value			Controlling for Marital Timing *p* Value		
B. Tests of Significance for Selected Contrasts (*p* values based on chi-square tests)							
Within education, across desired family size							
No high school diploma	0 or 1 child vs. 2 children	.01 *			.07 ^†^		
	3+ vs. 2 children	.18			.18		
High school diploma	0 or 1 child vs. 2 children	.23			.31		
	3+ vs. 2 children	.06 ^†^			.05 ^†^		
Some college	0 or 1 child vs. 2 children	.94			.79		
	3+ vs. 2 children	.66			.44		
Bachelor’s degree or higher	0 or 1 child vs. 2 children	.00 ***			.00 ***		
	3+ vs. 2 children	.19			.42		
Within desired family size, across education							
No high school diploma vs. bachelor’s degree	0 or 1 child	.13			.01 ^†^		
High school diploma vs. bachelor’s degree		.00 ***			.00 ***		
Some college vs. bachelor’s degree		.00 ***			.00 ***		
No high school diploma vs. bachelor’s degree	2 children	.02 *			.02 *		
High school diploma vs. bachelor’s degree		.24			.21		
Some college vs. bachelor’s degree		.75			.84		
No high school diploma vs. bachelor’s degree	3+ children	.09 ^†^			.17		
High school diploma vs. bachelor’s degree		.00 ***			.00 ***		
Some college vs. bachelor’s degree		.23			.83		

*Note: CI* = confidence interval.

^†^*p* < .10; *p < .05; ***p < .001

The incidence of motherhood is lowest among women with a bachelor’s degree or higher who desire no or one child: 64% of them will become mothers, compared with 79% of women without high school education and 83% of those with a high school diploma who desire no or one child. Differences in achieved motherhood between those desiring two children and those desiring three or more children are not statistically significant in any education group. Comparing across education groups, the most-educated women desiring three or more children are significantly less likely to become mothers than high school–educated women (*p* < .001) and marginally significantly less likely than women not graduating from high school (*p* < .10) and desiring three or more children. Among women who desire two children, only the difference between the highest and lowest education groups is statistically significant, driven by the outstandingly high motherhood rates of 95% of women who desire two children and did not graduate from high school.

#### Parity Among Parents

**Fig. 2:**
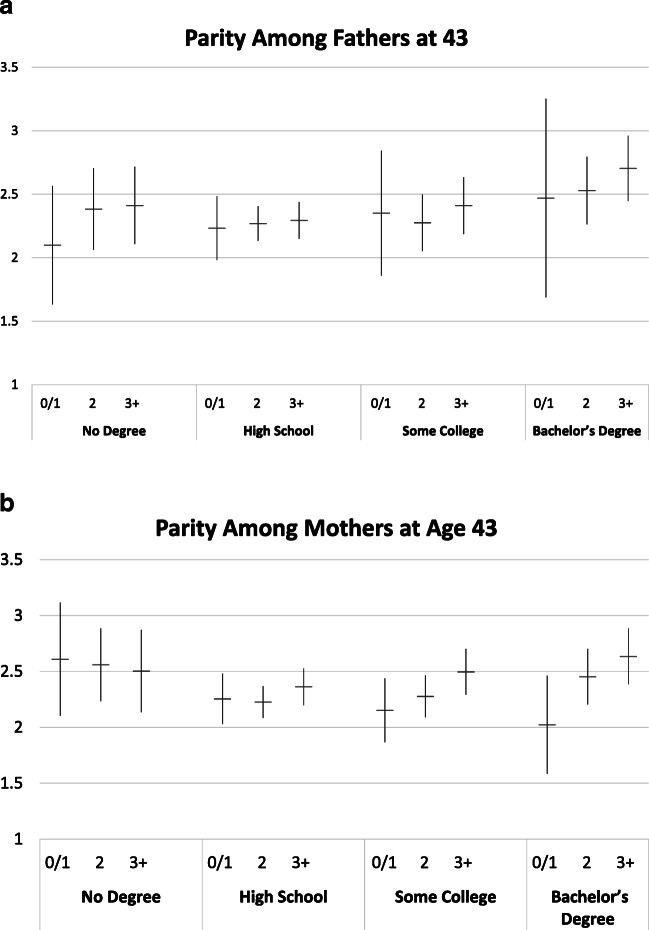
Predicted parity among fathers and mothers at age 43 by desired number of children in adolescence and completed education. Models control for race, Hispanic ethnicity, religiosity in 1979, family structure at age 14, number of siblings, and mother’s education.

Figure 2 depicts the average predicted number of children ever born to parents, by desired number of children and education. Unsurprisingly, in almost all groups, desiring more children is associated with higher average parity, conditional on being a parent, although confidence intervals overlap in many cases. What is less expected is that among the group of parents who desired two children or three or more children, both fathers with a bachelor’s degree and mothers with a bachelor’s degree have the *highest* average parity compared with the lower-educated. In fact, those with a bachelor’s degree who desire three or more children have the highest average parity of all groups, among both men and women. Women with no high school education who desire no or one child have similarly high parity, but they are a small group, and the confidence interval is wide.

#### Summary

Taken together, these results show little evidence of disproportional underachieving among highly educated men. They suggest that underachievement among highly educated women is primarily attributable to differences in the proportion of those who become a mother, not in parity differences among mothers. Desiring no or one child is associated with the lowest average parity among college-educated mothers only, but this low achieved parity is not underachieving, neither in our definition nor in the discrepancy measure definition.

### Marital Timing and Underachieving

**Fig. 3:**
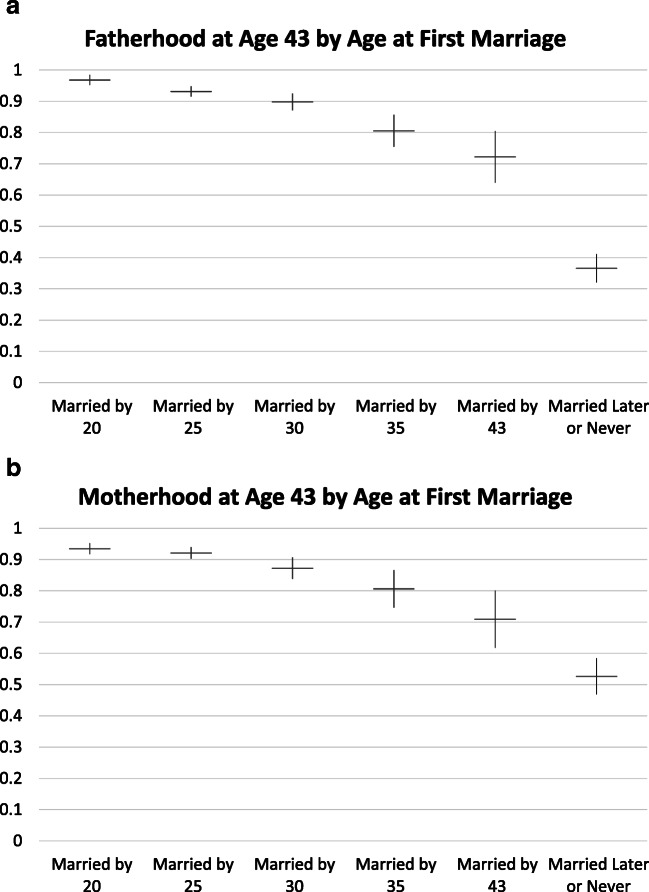
Predicted probability of fatherhood and motherhood by age at first marriage. Models control for race, Hispanic ethnicity, religiosity in 1979, family structure at age 14, number of siblings, mother’s education, completed education, and number of desired children.

**Fig. 4:**
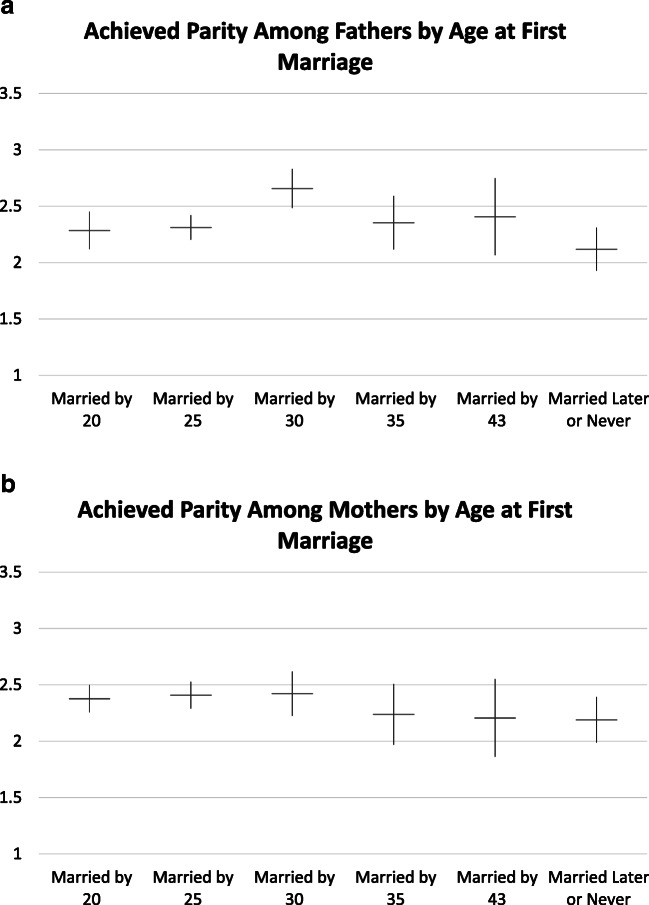
Predicted average parity among fathers and mothers by age at first marriage. Models control for race, Hispanic ethnicity, religiosity in 1979, family structure at age 14, number of siblings, mother’s education, completed education, number of desired children, and age at first birth.

In Figs. 3 and 4, we present adjusted predicted probabilities for parenthood and parity by age at marriage.

#### Entry Into Parenthood

Based on Fig. 3, parenthood probabilities clearly decline by age at first marriage, confirming H1. For both men and women, the largest decline is when marriage is postponed beyond age 30 to 35 (men, *p* < .01; women, *p* < .05). There is further decline for marriage postponed past age 35, but this decline is not statistically significant at conventional levels (men, *p* = .11; women, *p* = .11). Declines and levels are very similar for men and women, contrary to our expectations, rejecting H1a.

#### Parity Among Parents

Figure 4 shows no decline in parity with age at marriage up to age 30 for either fathers or mothers. For both mothers and fathers, parity declines with age at marriage after age 30, but declines are relatively small and are statistically significant only for fathers. Average parity is around 2 even for parents who marry late or never marry. Overall, Fig. 4 provides only weak support for H2 and no support for H2a.

#### Differential Associations by Achieved Education

**Fig. 5:**
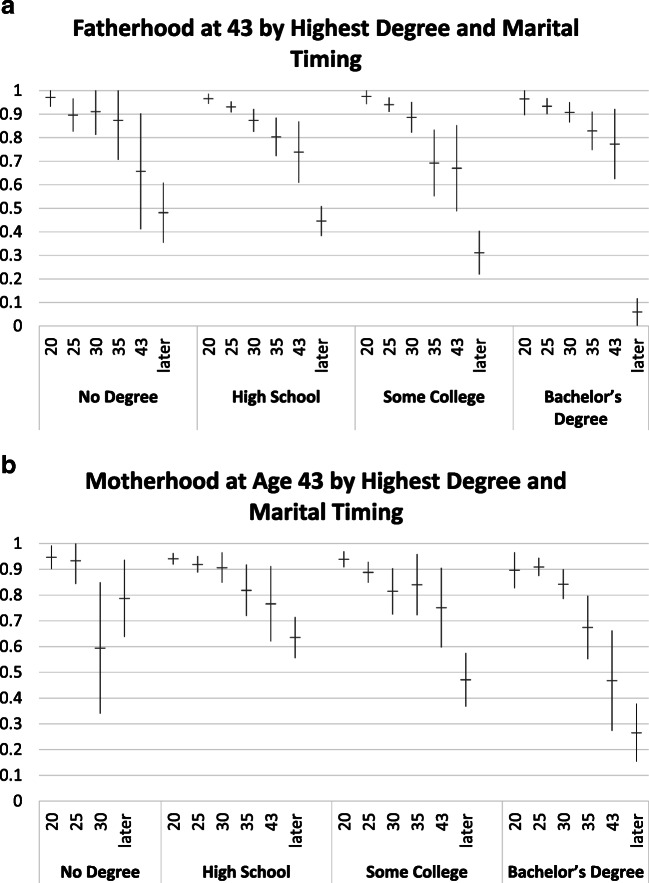
Fatherhood and motherhood at age 43 by highest degree and marital timing. Models control for race, Hispanic ethnicity, religiosity in 1979, family structure at age 14, number of siblings, mother’s education, completed education, and number of desired children.

**Fig. 6:**
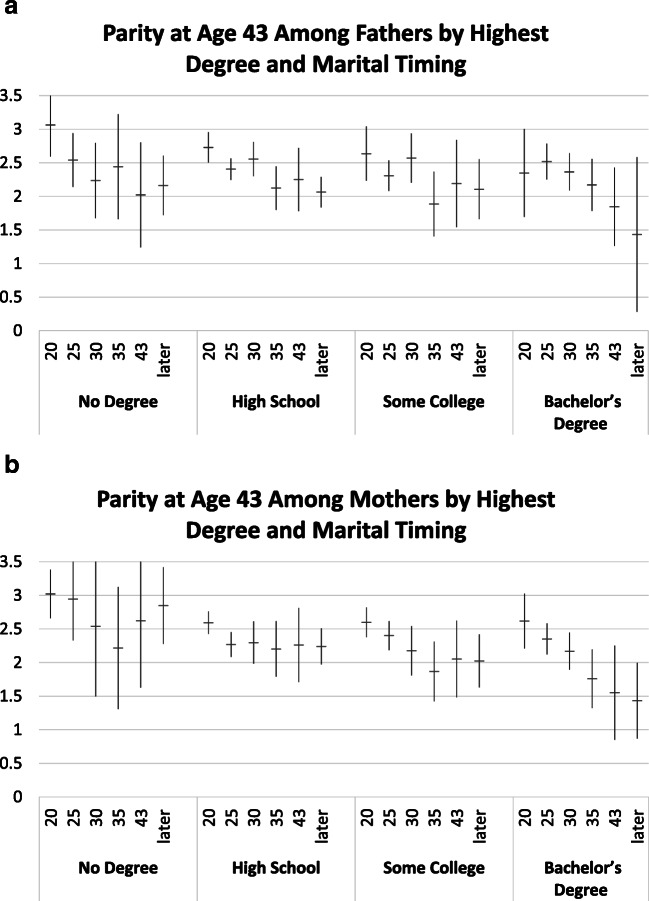
Average predicted parity among fathers and mothers by highest degree and marital timing. Models control for race, Hispanic ethnicity, religiosity in 1979, family structure at age 14, number of siblings, mother’s education, completed education, number of desired children, and age at first birth.

Results from models allowing for interactions with education are displayed in Figs. 5 and 6. The decline in parenthood with delayed marital timing is larger for the most-educated women but not men (Fig. 5). This partly confirms H1b, for women only. All models underlying the predicted probabilities control for desired family size. Hence, possible selection into marital timing by desired number of children should not drive our findings.

When achieved parity is broken down by age at marriage and by education (Fig. 6), the overall pattern shows a decline in average parity only up to age 35 for men and women without a college degree. For those with a bachelor’s degree or higher, completed parity continues to decline with age at marriage, to around 1.5 for those marrying past age 30. This pattern reflects a greater dependence on marriage for childbearing not only for parenthood but also for achieved parity among the most-educated men and women. Cell sizes are too small to differentiate further by desired number of children here, but results presented earlier suggest possible differences by desired parity. H2b is thus only partly confirmed; parity declines for the most-educated are more pronounced with increasing age at marriage. This suggests that competing devotions, changing preferences, or social constraints may shape family size as well as entry into parenthood when marriage occurs late. However, those not yet married at age 35 are a rather small group in this cohort (see Table 1 for descriptive statistics). Some of them may subsequently form unions with individuals who already are parents, thus removing or satisfying their desire for own children, or downward adjusting the number of desired children.

### Testing for the Mediating Effect of Marital Timing

Tables 2 and 3 illustrate predicted probabilities of fatherhood and motherhood by education and desired parity, before and after controlling for marital timing (see panel A in the tables). (The probabilities before controlling for marital timing are the same as those used to generate Fig. 1 presented earlier, corresponding to Tables A1 and A2 in the online appendix). Figures 2 and 4 illustrate that educational differences in underachieving among women hinge on differential chances of motherhood—not on differences in parity among mothers. Therefore, we show results of testing the mediating effect of marital timing only on parenthood, not on parity. We test whether the predicted probabilities of parenthood are significantly different from each other (1) within education groups across desired parity or (2) within desired parity groups across education, before and after we control for marital timing. Test results are shown in panel B of the tables. We focus our discussion on differences for women with a bachelor’s degree and relatively large desired family size, the group for which underachievement is most marked.

As discussed earlier (see Fig. 1), there are only a few significant differences in achieving fatherhood either by education (within desired parity) or by desired parity (within education). These differences are only slightly attenuated after we control for marital timing, and they remain at least marginally statistically significant.

There were more differences by desired number of children and education in parenthood chances among women than among men (Fig. 2). Most notably, among women desiring three or more children, women with a bachelor’s degree or higher are least likely to become parents in the models not accounting for marital timing. This difference is statistically significant for the contrast with high school–educated women (*p* < .001) and marginally significant for the contrast with women without high school education (*p* = .09). Some of these differences are reduced in models controlling for age at marriage. After age at marriage is accounted for, the difference between college-educated women and women without a high school diploma is insignificant (*p* = .17), yet the difference with women with high school education remains statistically significant.

The mediation analysis supports these results. Indirect effects, operating through differences in marital timing, are marginally significant for the difference with women without a high school diploma (*p* = .08) but are not statistically significant for differences with women with a high school education. The latter difference in motherhood hinges on the direct effect of fertility desires (*p* < .001). Interestingly, a significant indirect effect (marital timing) on motherhood differences is present between college-educated women desiring three and more children and college-educated women desiring two children (*p* = .02) as well as women with some college desiring three or more children (*p* = .01). Marital timing thus plays some role in motherhood differences between the most-educated women desiring three or more children and their lower-educated counterparts. However, it does not appear to be driving the educational difference in motherhood among women who desire three or more children, and thus it does not seem to be a key contributor to education differences in underachievement.

### Summary: The Role of Marital Timing

In this study, we ask and answer two questions about the role of marital timing—and specifically the postponement of marriage—in explaining fertility differences by education and the underachievement of fertility desires. Table 4 summarizes our main findings.

**Table 4:** Summary of findings

Outcome	Significant Education Differences in Achieved Fertility When Not Controlling for Marriage? (Table 2, Table 3)	Significant Education Differences in Achieved Fertility After Controlling for Marriage? (Table 2, Table 3)	Significant Association Between Marriage Timing and Achieved Fertility? (Fig. 3, Fig. 4)
Fatherhood	**Mostly not:** only for high school vs. bachelor’s degree or more among those desiring two children	**Mostly not:** high school vs. bachelor’s degree or more, desiring two children now marginally significant; no high school vs. bachelor’s degree or more, 3+ children, now larger and significant	**Yes:** those who marry later much less likely to become fathers; no variation in association by education
Motherhood	**Yes:** for most contrasts, women with a bachelor’s degree or more less likely to become mothers than their less-educated counterparts with the same desired family size	**Yes:** most contrasts still statistically significant and similar in magnitude	**Yes:** those who marry later much less likely to become mothers; strongest association for most-educated women
Parity Among Fathers	**Hardly:** Achieved fertility slightly higher among most-educated, if anything	**Not tested**	**Little difference** in full sample (no clear trend, all within confidence interval); some suggestion of larger impact for most-educated
Parity Among Mothers	**Not really:** among women desiring 0/1, achieved fertility lower for women with a bachelor’s degree or more than for less-educated counterparts; otherwise, overlapping confidence intervals	**Not tested**	**Little difference** in full sample; slightly lower achieved parity for married at 30+, but overlapping CIs; some suggestion of larger impact for most-educated

*Notes:* Data are for men and women aged 18 or older at the first interview, observed at least once at age 43 or older, with nonmissing data on key dependent and independent variables. See Tables 2 and 3 and Figs. 3 and 4 for details.

*Source:* National Longitudinal Survey of Youth, 1979 cohort.

First, we ask whether marriage postponement explains lower fertility among more highly educated men and women. Here, the answer is a qualified “no.” The most consistent educational difference in fertility uncovered in our analysis is the difference in the proportion of women with a bachelor’s degree who become mothers relative to their less-educated counterparts. Education differences for fatherhood and education differences in achieved parity among parents are small and generally not statistically significant. Although these differences are somewhat attenuated by controlling for marriage timing in some education by desired family size comparisons (detailed in the Discussion and Conclusion section), they largely remain even after accounting for marriage postponement. Thus, marriage postponement does not explain the high levels of underachievement of fertility desires among college-educated women relative to lower-educated women with the same number of desired children.

Second, we ask whether marriage postponement matters for fertility. Here, the answer is a clear “yes.” Both men and women who marry later or not at all are less likely to become parents than their counterparts who marry earlier. For women, this association is strongest among the most-educated. For achieved parity among parents, the association with marriage timing is weaker, but there is some suggestion that the most-educated mothers and fathers have smaller completed family size if they marry later.

## Discussion and Conclusion

In this study, we investigate the underachievement of early fertility desires in the NLSY79 cohort among men and women, with a focus on differences by education and desired parity and whether this association differs between men and women and across education groups. We furthermore extend the literature by differentiating underachieving into two components: namely, in the achievement of parenthood and the achievement of completed parity among those who became parents.

Two main results come to the fore. First, we find that education differences in underachieving vary by desired family size among women but not men and that these differences by desired number of children hinge mainly on chances to achieve motherhood, not on the number of children conditional on having any children. The most-educated women desiring three or more children are more likely to remain childless, not only compared with lower-educated women desiring three or more children but also compared with their highly educated peers desiring two children (even though this last contrast between college-educated women just misses statistical significance). Yet, these women have the highest parity among all women if they do become mothers. Given that almost one-half of all women with a bachelor’s degree or higher said they were aspiring to become mothers of three or more children, this is a remarkable finding. It appears that a significant proportion of the educational fertility difference among women may hinge upon women who desired many children, completed a bachelor’s degree, and subsequently remained childless.

Multiple pathways may explain this pattern, including changing fertility desires, work-family conflict leading to forgone motherhood, or differences in the meaning of early fertility desires as well as the agency to achieve these desires between social groups. Future research is needed to investigate those pathways, specifically with regard to deeper investigations into the meaning of measurements of fertility desires and intentions. Although a large empirical literature has examined linkages between fertility intentions or desires and behavior, conceptual reflections and empirical examinations of the underlying constructs that these indicators measure, as well whether their meaning may differ across social groups (e.g., Philipov and Bernardi 2012), have been more rare yet seem called for.

In our second main result, we show that postponing marriage past the age of 30 is associated with declines in parenthood rates and, albeit to a lesser extent, achieved parity among parents. This association is strongest among the most-educated, who tend to not have children outside of marriage. After desired family size is controlled for, men who do not marry before the age of 43 are the most likely to remain childless, particularly among those who are highly educated. Overall, the relationship between marriage timing and fertility is remarkably similar for women and men. This similarity is surprising, given our hypothesis that marital delay would affect men’s fertility less than women’s because they tend to marry younger women and are thought to experience slower declines in fecundity with age. We believe that our findings underscore the importance of social factors for childbearing processes and hint at a perhaps less important role of biology.

Our study is not without limitations. While offering a new conceptualization of underachieving, we group those desiring small families and those desiring large families into only two categories, losing detail. We believe that pooling cases in this way is meaningful, yet precision is lost in terms of understanding whether women desiring one child may underachieve more often among the college-educated than less-educated women and whether underachieving motherhood among college-educated women desiring large families may hinge particularly on women who want more than three children. Also, as discussed earlier, life courses are long, and fertility and other goals are malleable. We avoid working with intentions or desires measured later in life to overcome the issue that they have been already affected by other experiences (e.g., lack of partner), but we thereby also miss measuring changes in desired fertility that are not triggered by constraints to childbearing. Future research is needed to investigate whether people who initially want large families but later remain childless may have revised their life plans to embark on other paths than previously envisioned. This includes an investigation into potential systematic differences in the meaning of fertility desires among adolescents, which could also in part be behind our findings. For instance, fertility desires may represent more immediate goals among those who are not planning to go to college and are expecting to have children early in life, whereas they may reflect more abstract ideas of distant future lives among the college-bound. In addition, ideation of the importance of motherhood has been shown to mediate the relationship between social background and fertility intentions (McQuillan et al. 2015). It is possible that motherhood ideation systematically varies among those who will become highly educated and those who will not, potentially mediating whether and when fertility desires translate into childbearing behavior. And if there is larger variation in the importance of parenthood ideation between men and women, particularly by projected education trajectories, this might be a factor behind the gender differences in the fertility desire–parenthood association we find.

Nonetheless, our findings bring together the literature on education differences in fertility and the literature on underachieving fertility desires. We show that childlessness—not small family size among parents—is the main contributor of underachieving among U.S. women with a bachelor’s degree or higher. Hence, childlessness emerges as the main driver not only of the education fertility difference but also of how fertility desires are mediated by education in their translation into different life courses. Studies examining the consequences of unrealized fertility on other life outcomes—for example, on well-being in old age—have primarily been concerned with the effects of childlessness (e.g., involuntary childlessness) or of the experience of parenting as such (Chou and Chi 2004; Maximova and Quesnel-Vallée 2009; Zhang and Hayward 2001; Zhang and Liu 2007; for a review, see Umberson et al. 2010). These findings imply that the dichotomy of remaining childless versus becoming a parent is perceived as potentially more consequential than parity or than having one or two children less than desired (among those who become parents), at least by the research community. This further underscores the need to differentiate underachieving into a parenthood and a parity component.

We also note that women with a bachelor’s degree or higher who wanted no or one child were much more likely to remain childless compared with both their lower-educated counterparts and college-educated women who desired more children, and that this was not a function of marital timing differences. (This pattern does not hold for men.) This finding is less relevant for underachievement (although some women in this group will have underachieved their desire for one child) but is important for understanding the larger question of how women implement early fertility desires. Contrary to Lutz’s (2017:27) argument that “education typically empowers women (and couples) to reach their personal target for family size, regardless of what the target is,” our results suggest that a bachelor’s degree may provide women with the agency to realize their desires for *small* families more than their desires for *large* families, at least in this U.S. birth cohort. It remains for future research to uncover how the education agency issue plays out across different places and times. In particular, the agency (and economic resources) provided by education may be more salient in contexts where infertility treatments are more effective and more widely available than they were for this cohort.

Keizer et al. (2008:873) concluded that “childlessness debates require a shift in focus. Concerns about the incompatibility of work with caring tasks need to be supplemented with concerns about entering and remaining in partnerships.” Yet, an emphasis to analytically integrate partnership and childbearing trajectories to understand fertility in general and childlessness in particular is only starting to come to the fore more formally (Trimarchi and Van Bavel 2017). Our findings underscore the important role that union formation chances and timing play for childlessness, particularly among highly educated women who originally desired large families or highly educated men forming first coresidential unions late in life.

## Electronic supplementary material

ESM 1 (DOCX 38 kb)

## Data Availability

Data from the National Longitudinal Survey of Youth, 1979 (NLSY79) are used for all analyses. They are publicly available on the NLSY website: www.nlsinfo.org/content/cohorts/nlsy79.

## Footnotes

1 Racial groups other than Black or White are too small for separate analysis and are therefore excluded.
