# Supplementary material for: Preferences, Partners, and Parenthood: Linking Early Fertility Desires, Marriage Timing, and Achieved Fertility
Source: Demography. 2020 Nov 12;57(6):1975–2001. doi: 10.1007/s13524-020-00927-y (PMC7732806; doi:10.1007/s13524-020-00927-y)
Supplement: Supplementary file 1 — (DOCX 38 kb) [file 13524_2020_927_MOESM1_ESM.docx]

**Online Appendix**

Table A1: Logistic regression models predicting fatherhood at age 43 by completed educational degree and desired number of children, without (model 1) and with (model 2) controls for marital timing.

|  | **Model 1** | | | **Model 2** | | |
| --- | --- | --- | --- | --- | --- | --- |
|  | **Coef.** | **SE** | **p** | **Coef.** | **SE** | **p** |
| Desired family size x educational attainment category (reference = 2 children, high school degree) |  |  |  |  |  |  |
| 0/1, no hs degree | -0.43 | 0.37 | 0.24 | -0.42 | 0.42 | 0.32 |
| 2, no hs degree | -0.13 | 0.28 | 0.64 | -0.20 | 0.32 | 0.53 |
| 3+, no hs degree | -0.13 | 0.27 | 0.62 | 0.34 | 0.31 | 0.28 |
| 0/1, hs degree | -0.63 | 0.20 | 0.00 | -0.48 | 0.24 | 0.05 |
| 3+, hs degree | -0.19 | 0.16 | 0.23 | -0.07 | 0.19 | 0.72 |
| 0/1, some college | -0.23 | 0.38 | 0.54 | -0.30 | 0.45 | 0.50 |
| 2 some college | -0.30 | 0.19 | 0.12 | -0.41 | 0.23 | 0.07 |
| 3+ some college | -0.35 | 0.20 | 0.07 | -0.36 | 0.23 | 0.11 |
| 0/1, BA or more | -0.46 | 0.46 | 0.31 | -0.17 | 0.54 | 0.75 |
| 2, BA or more | -0.44 | 0.20 | 0.03 | -0.41 | 0.23 | 0.08 |
| 3+, BA or more | -0.37 | 0.19 | 0.06 | -0.37 | 0.23 | 0.10 |
| Hispanic (vs non-Hispanic) | 0.12 | 0.14 | 0.42 | 0.43 | 0.17 | 0.01 |
| Black (vs non-Black) | 0.28 | 0.12 | 0.03 | 1.32 | 0.16 | 0.00 |
| Lived with both parents at 14 | 0.02 | 0.11 | 0.86 | -0.09 | 0.13 | 0.51 |
| Frequency of religious attendance | 0.03 | 0.03 | 0.31 | 0.00 | 0.04 | 0.99 |
| Number of siblings | 0.10 | 0.06 | 0.09 | 0.11 | 0.07 | 0.12 |
| Mother’s education (reference = no high school degree) |  |  |  |  |  |  |
| High school degree | -0.19 | 0.12 | 0.12 | -0.28 | 0.14 | 0.05 |
| Some college | 0.13 | 0.20 | 0.51 | 0.22 | 0.23 | 0.34 |
| Bachelor’s degree or more | -0.03 | 0.22 | 0.89 | 0.06 | 0.25 | 0.80 |
| Missing | -0.12 | 0.21 | 0.56 | -0.05 | 0.24 | 0.85 |
| Age at marriage (reference = before age 25) |  |  |  |  |  |  |
| By age 25 |  |  |  | -0.80 | 0.29 | 0.01 |
| By age 30 |  |  |  | -1.24 | 0.31 | 0.00 |
| By age 35 |  |  |  | -1.99 | 0.32 | 0.00 |
| By age 43 |  |  |  | -2.45 | 0.34 | 0.00 |
| Later or never |  |  |  | -3.96 | 0.29 | 0.00 |
| Constant | 1.20 | 0.23 | 0.00 | 2.98 | 0.37 | 0.00 |

Data: National Longitudinal Survey of Youth, 1979 cohort. N=2589 men age 18 or older at the first interview, observed at least once at age 43 or older, with non-missing data on key dependent and independent variables.

Table A2: Logistic regression models predicting motherhood at age 43 by completed educational degree and desired number of children, without (model 3) and with (model 4) controls for marital timing.

|  | **Model 3** | | | **Model 4** | | |
| --- | --- | --- | --- | --- | --- | --- |
|  | **Coef.** | **SE** | **p** | **Coef.** | **SE** | **p** |
| Desired family size x educational attainment category (reference = 2 children, high school degree) |  |  |  |  |  |  |
| 0/1, no hs degree | -0.54 | 0.42 | 0.20 | -0.14 | 0.44 | 0.75 |
| 2, no hs degree | 1.06 | 0.53 | 0.05 | 1.06 | 0.55 | 0.05 |
| 3+, no hs degree | 0.19 | 0.42 | 0.66 | 0.15 | 0.45 | 0.74 |
| 0/1, hs degree | -0.28 | 0.23 | 0.23 | -0.26 | 0.25 | 0.31 |
| 3+, hs degree | 0.42 | 0.22 | 0.06 | 0.47 | 0.24 | 0.05 |
| 0/1, some college | -0.21 | 0.28 | 0.46 | -0.16 | 0.30 | 0.60 |
| 2 some college | -0.19 | 0.21 | 0.37 | -0.25 | 0.22 | 0.26 |
| 3+ some college | -0.28 | 0.21 | 0.18 | -0.43 | 0.23 | 0.05 |
| 0/1, BA or more | -1.24 | 0.27 | 0.00 | -1.42 | 0.30 | 0.00 |
| 2, BA or more | -0.26 | 0.22 | 0.24 | -0.30 | 0.24 | 0.21 |
| 3+, BA or more | -0.54 | 0.21 | 0.01 | -0.48 | 0.22 | 0.03 |
| Hispanic (vs non-Hispanic) | 0.06 | 0.17 | 0.73 | 0.35 | 0.18 | 0.05 |
| Black (vs non-Black) | -0.10 | 0.14 | 0.48 | 0.65 | 0.16 | 0.00 |
| Lived with both parents at 14 | -0.27 | 0.13 | 0.04 | -0.31 | 0.14 | 0.02 |
| Frequency of religious attendance | 0.03 | 0.03 | 0.44 | -0.01 | 0.04 | 0.89 |
| Number of siblings | 0.14 | 0.06 | 0.03 | 0.18 | 0.07 | 0.01 |
| Mother’s education (reference = no high school degree) |  |  |  |  |  |  |
| High school degree | -0.30 | 0.14 | 0.03 | -0.25 | 0.15 | 0.09 |
| Some college | -0.36 | 0.21 | 0.08 | -0.17 | 0.22 | 0.44 |
| Bachelor’s degree or more | -0.48 | 0.22 | 0.03 | -0.27 | 0.24 | 0.25 |
| Missing | 0.08 | 0.30 | 0.79 | 0.27 | 0.32 | 0.41 |
| Age at marriage (reference = before age 25) |  |  |  |  |  |  |
| By age 25 |  |  |  | -0.21 | 0.19 | 0.28 |
| By age 30 |  |  |  | -0.74 | 0.22 | 0.00 |
| By age 35 |  |  |  | -1.24 | 0.24 | 0.00 |
| By age 43 |  |  |  | -1.77 | 0.27 | 0.00 |
| Later or never |  |  |  | -2.56 | 0.19 | 0.00 |
| Constant | 1.20 | 0.23 | 0.00 | 2.98 | 0.37 | 0.00 |

Data: National Longitudinal Survey of Youth, 1979 cohort. N=2566 women age 18 or older at the first interview, observed at least once at age 43 or older, with non-missing data on key dependent and independent variables.

Table A3: General linear models (Poisson distribution, ml optimization) predicting parity at age 43 among fathers by completed educational degree and desired number of children, without (model 5) and with (model 6) controls for marital timing.

|  | **Model 5** | | | **Model 6** | | |
| --- | --- | --- | --- | --- | --- | --- |
|  | **Coef.** | **SE** | **p** | **Coef.** | **SE** | **p** |
| Desired family size x educational attainment category (reference = 2 children, high school degree) |  |  |  |  |  |  |
| 0/1, no hs degree | -0.08 | 0.12 | 0.51 | -0.07 | 0.12 | 0.56 |
| 2, no hs degree | 0.05 | 0.07 | 0.50 | 0.05 | 0.07 | 0.46 |
| 3+, no hs degree | 0.06 | 0.07 | 0.39 | 0.07 | 0.07 | 0.35 |
| 0/1, hs degree | -0.02 | 0.06 | 0.81 | -0.03 | 0.06 | 0.70 |
| 3+, hs degree | 0.01 | 0.04 | 0.80 | 0.01 | 0.04 | 0.75 |
| 0/1, some college | 0.04 | 0.11 | 0.75 | 0.04 | 0.11 | 0.75 |
| 2 some college | 0.00 | 0.06 | 0.96 | -0.01 | 0.06 | 0.93 |
| 3+ some college | 0.06 | 0.06 | 0.28 | 0.06 | 0.06 | 0.32 |
| 0/1, BA or more | 0.09 | 0.17 | 0.61 | 0.06 | 0.17 | 0.72 |
| 2, BA or more | 0.11 | 0.06 | 0.09 | 0.08 | 0.06 | 0.20 |
| 3+, BA or more | 0.18 | 0.06 | 0.00 | 0.15 | 0.06 | 0.01 |
| Age at first birth | -0.03 | 0.00 | 0.00 | -0.04 | 0.00 | 0.00 |
| Hispanic (vs non-Hispanic) | 0.08 | 0.04 | 0.04 | 0.08 | 0.04 | 0.04 |
| Black (vs non-Black) | 0.00 | 0.04 | 0.96 | 0.01 | 0.04 | 0.83 |
| Lived with both parents at 14 | 0.00 | 0.03 | 0.94 | 0.00 | 0.03 | 0.92 |
| Frequency of religious attendance | 0.00 | 0.01 | 0.63 | 0.00 | 0.01 | 0.63 |
| Number of siblings | 0.01 | 0.02 | 0.65 | 0.01 | 0.02 | 0.78 |
| Mother’s education (reference = no high school degree) |  |  |  |  |  |  |
| High school degree | -0.04 | 0.04 | 0.30 | -0.04 | 0.04 | 0.24 |
| Some college | -0.02 | 0.06 | 0.77 | -0.02 | 0.06 | 0.68 |
| Bachelor’s degree or more | 0.00 | 0.07 | 0.99 | -0.01 | 0.07 | 0.94 |
| Missing | -0.10 | 0.06 | 0.10 | -0.10 | 0.06 | 0.09 |
| Age at marriage (reference = before age 25) |  |  |  |  |  |  |
| By age 25 |  |  |  | 0.01 | 0.04 | 0.78 |
| By age 30 |  |  |  | 0.15 | 0.05 | 0.00 |
| By age 35 |  |  |  | 0.03 | 0.07 | 0.66 |
| By age 43 |  |  |  | 0.05 | 0.08 | 0.54 |
| Later or never |  |  |  | -0.08 | 0.06 | 0.20 |
| Constant | 1.67 | 0.10 | 0.00 | 1.72 | 0.10 | 0.00 |

Data: National Longitudinal Survey of Youth, 1979 cohort. N=2037 fathers age 18 or older at the first interview, observed at least once at age 43 or older, with non-missing data on key dependent and independent variables.

Table A4: General linear models (Poisson distribution, ml optimization) predicting parity at age 43 among mothers by completed educational degree and desired number of children, without (model 7) and with (model 8) controls for marital timing.

|  | **Model 7** | | | **Model 8** | | |
| --- | --- | --- | --- | --- | --- | --- |
|  | **Coef.** | **SE** | **p** | **Coef.** | **SE** | **p** |
| Desired family size x educational attainment category (reference = 2 children, high school degree) |  |  |  |  |  |  |
| 0/1, no hs degree | 0.16 | 0.10 | 0.12 | 0.18 | 0.10 | 0.08 |
| 2, no hs degree | 0.14 | 0.07 | 0.05 | 0.15 | 0.07 | 0.04 |
| 3+, no hs degree | 0.12 | 0.08 | 0.14 | 0.12 | 0.08 | 0.13 |
| 0/1, hs degree | 0.01 | 0.06 | 0.83 | 0.01 | 0.06 | 0.87 |
| 3+, hs degree | 0.06 | 0.05 | 0.20 | 0.06 | 0.05 | 0.21 |
| 0/1, some college | -0.03 | 0.08 | 0.66 | -0.03 | 0.08 | 0.65 |
| 2 some college | 0.02 | 0.05 | 0.67 | 0.02 | 0.05 | 0.73 |
| 3+ some college | 0.11 | 0.05 | 0.03 | 0.11 | 0.05 | 0.05 |
| 0/1, BA or more | -0.10 | 0.12 | 0.41 | -0.11 | 0.12 | 0.35 |
| 2, BA or more | 0.10 | 0.06 | 0.12 | 0.09 | 0.06 | 0.18 |
| 3+, BA or more | 0.17 | 0.06 | 0.01 | 0.16 | 0.06 | 0.01 |
| Age at first birth | -0.03 | 0.00 | 0.00 | -0.03 | 0.00 | 0.00 |
| Hispanic (vs non-Hispanic) | 0.05 | 0.04 | 0.16 | 0.06 | 0.04 | 0.13 |
| Black (vs non-Black) | -0.02 | 0.04 | 0.62 | 0.01 | 0.04 | 0.85 |
| Lived with both parents at 14 | 0.00 | 0.03 | 0.89 | -0.01 | 0.03 | 0.82 |
| Frequency of religious attendance | 0.01 | 0.01 | 0.13 | 0.01 | 0.01 | 0.17 |
| Number of siblings | 0.05 | 0.02 | 0.00 | 0.05 | 0.02 | 0.00 |
| Mother’s education (reference = no high school degree) |  |  |  |  |  |  |
| High school degree | -0.01 | 0.03 | 0.71 | -0.01 | 0.03 | 0.71 |
| Some college | 0.06 | 0.06 | 0.33 | 0.06 | 0.06 | 0.33 |
| Bachelor’s degree or more | 0.07 | 0.07 | 0.29 | 0.07 | 0.07 | 0.29 |
| Missing | 0.03 | 0.06 | 0.59 | 0.03 | 0.06 | 0.59 |
| Age at marriage (reference = before age 25) |  |  |  |  |  |  |
| By age 25 |  |  |  | 0.01 | 0.04 | 0.72 |
| By age 30 |  |  |  | 0.02 | 0.05 | 0.71 |
| By age 35 |  |  |  | -0.06 | 0.07 | 0.38 |
| By age 43 |  |  |  | -0.07 | 0.08 | 0.38 |
| Later or never |  |  |  | -0.08 | 0.05 | 0.13 |
| Constant | 1.40 | 0.09 | 0.00 | 1.40 | 0.10 | 0.00 |

Data: National Longitudinal Survey of Youth, 1979 cohort. N=2147 mothers age 18 or older at the first interview, observed at least once at age 43 or older, with non-missing data on key dependent and independent variables.
